# Expected goals in football: Improving model performance and demonstrating value

**DOI:** 10.1371/journal.pone.0282295

**Published:** 2023-04-05

**Authors:** James Mead, Anthony O’Hare, Paul McMenemy

**Affiliations:** Computing Science and Mathematics, University of Stirling, Stirling, United Kindom; Universiti Malaysia Terengganu, MALAYSIA

## Abstract

Recently, football has seen the creation of various novel, ubiquitous metrics used throughout clubs’ analytics departments. These can influence many of their day-to-day operations ranging from financial decisions on player transfers, to evaluation of team performance. At the forefront of this scientific movement is the metric *expected goals*, a measure which allows analysts to quantify how likely a given shot is to result in a goal however, xG models have not until this point considered using important features, e.g., player/team ability and psychological effects, and is not widely trusted by everyone in the wider football community. This study aims to solve both these issues through the implementation of machine learning techniques by, modelling expected goals values using previously untested features and comparing the predictive ability of traditional statistics against this newly developed metric. Error values from the expected goals models built in this work were shown to be competitive with optimal values from other papers, and some of the features added in this study were revealed to have a significant impact on expected goals model outputs. Secondly, not only was expected goals found to be a superior predictor of a football team’s future success when compared to traditional statistics, but also our results outperformed those collected from an industry leader in the same area.

## Introduction

Uncertainty plays a role in all sports and is a key reason why people enjoy interacting with it. The knowledge that luck (alongside performance) can determine who wins and loses is what draws many people in. This factor is arguably most prevalent in football. Due to its low-scoring nature when compared to other sports, uncertainty often highly influences the result of a match [1–5]. This is the ultimate motivation behind novel metrics such as expected goals (commonly shortened to ‘xG’). Put simply, expected goals assigns a probability between 0 and 1 to each shot taken by a team in a game (0 indicating no possibility of the shot being a goal and 1 indicating a definite goal). This is a better way of dealing with the randomness in football than, for example, a traditional goal-based metric since a shot is a much more common event than a goal [4, 5]. Producing a probability value, indicating how likely the shot is to result in a goal, helps to give analysts an unbiased view of what occurred in the game—more specifically, how many goals both teams ‘should have’ scored given the chances they created.

In 2018, FIFA reported that the most recent World Cup tournament in Russia amassed a viewership of 3.572 billion [6]. This figure dwarfs those reached in cricket—widely believed to be the second most popular sport, with audience estimates for the ICC Men’s Cricket World Cup in 2019 standing at 1.6 billion [7]. Naturally, this immense following means there is considerable economic value inherent within football. Therefore, discovering ways in which clubs are able to predict future outcomes with greater confidence and thus gain an advantage, can prove to be extremely financially beneficial. Expected goals provides analysts with this advantage, one which can aid in decision-making at both the sport-level and business-level of football. Not only can it help to improve the fortunes of football clubs on the pitch through tactical analysis of player and team performance, but it can also assist in financial situations such as player acquisition and contract negotiation. This is where xG’s true power lies.

Since xG’s inception, the metric has become ubiquitous within football. The majority of top-level football teams and betting companies make use of the statistic (and related concepts of expected assists and post-shot expected goals), with it aiding the development or acquisition of players in clubs and refinement of betting odds modelling for gambling sites [4, 8, 9]. Despite analytics teams at football clubs and statisticians at betting companies championing the idea of expected goals and even incorporating it into the work they do, the concept isn’t so widely regarded by fans and pundits. This paper will also aim to prove the value that expected goals can bring in football analytics, through comparing its predictability of match outcome against traditional methods.

It is not clear when the expected goals statistic was first developed and who conceived it, with most [1, 9, 10] stating that Macdonald’s [11] study into shot outcome in ice hockey originated the term, whilst others [3] have attributed it to Green’s [12] article. At its core, the concept of expected goals can be thought of as a classification problem (due to it being a probability of a shot being on target) this is why, in order to calculate these probabilities, machine learning and statistical methods are applied. Different approaches to modelling xG include logistic regression, gradient boosting, neural networks, support vector machines and tree-based classification algorithms [1, 2, 13]. Most of the features incorporated into these models are engineered from in-game data, split into two sections—event and positional. Event data comprises detailed information about all events which occur on the pitch during a match such as passes, duels, fouls, shots, etc. Each data point usually includes where the event took place on the pitch (*x* and *y* coordinates), where the event finished on the pitch (for shots, passes, etc.), which player was involved in the event, which match the event occurred in, whether the action was successful or not, and many other variables. These data points are manually tagged by a team of people watching the game. Positional data provides information on the location of every player, the referee and the ball with a frequency of up to 25 Hz [2]. This is compiled using either vision-based systems, GPS technology or radio wave-based tracking systems [14]. Unfortunately, both football event and positional data are rarely publicly available [1, 15, 16], with companies such as Opta, Wyscout and StatsBomb collecting the data themselves and selling it to football teams, betting firms or websites directly. This has therefore negatively impacted the depth of research surrounding the topic of expected goals due to the fact that both positional and event data (a combination which is incredibly rare to find) help create robust models with powerful features. Consequently, one objective of this paper is to add to the limited pool of literature on the subject of expected goals.

Since the purpose of expected goals models is to predict the likelihood that a given shot will result in a goal the predominant features are distance, angle and shot type. One flaw with models which incorporate only these features (or similar ones) is that they tend to be poor predictors for both above- and below-average teams [17]. Therefore, integrating important factors such as player/team ability and psychological effects into expected goals models is key to enhancing their performance. However, since it is difficult to create proxies which capture the impact of these factors, their inclusion is complicated. Hence, another area this paper aims to explore is the lack of certain factors which could influence the outcome of a shot, through engineering features representing player ability, team quality and psychological pressure, alongside more common features. Additionally, since separate models for separate leagues will be built, this will allow for evaluation of how the importance of certain features can vary between competitions and therefore determine the reasons as to why national leagues are subtly different to each other.

Expected goals models are primarily characterised by what features they include in order to ascertain the probability of a shot resulting in a goal. Shot location is the most common of such features and forms the basis of most models. This is usually incorporated through two variables − the distance from and angle to the goal when the shot was taken. Rathke’s [17] study integrated these variables into his model using data from the 2012/13 Premier League and Bundesliga seasons. He achieved this by splitting the football pitch into eight zones and analysing goal probability from shots in each one. Rathke found that both distance and angle significantly impact the likelihood of a goal being scored. Similarly, Spearman [3] examined the effect of distance and angle on shooting outcome. The model that Spearman built, using event data from a 14-team professional football league during the 2017/18 season, differed slightly from the norm due to the fact that he used a probabilistic approach to quantify what he calls ‘off-ball scoring opportunities’, or OBSO for short. As the name suggests, this tackled the issue of rewarding clever movement from players who never receive the ball, something which traditional expected goals models do not address. In spite of this difference, Spearman also found that location is an important feature to include in his model. Naturally, due to its salience when explaining the randomness of goal probability, shot location is discussed in almost all studies surrounding the topic of expected goals [2, 4, 13, 15, 18–20].

Another prevalent feature discussed in the literature is shot type. This feature provides contextual information about the shot and can be divided into two separate subfeatures. The first examines which part of the body is used when taking the shot ((left/right) foot, head, other). The second looks at what game situation the shot occurred in. This can include open play, counter attack, free kick and even penalty kick, depending on the model. As part of the model Brechot and Flepp [4] built in their work, they included these features and determined that both influence shot outcome. In particular, they found that a shot deriving from a free kick is more likely to be a goal, from a penalty kick even more so and from a header significantly less so, when compared to a shot taken in open play with either foot. Lucey et al.’s [19] study also incorporated match context into their expected goals model. They used data from an anonymous 20-team league comprising almost 10,000 shots and examined spatiotemporal patterns within the ten-second window before each shot event. When estimating goal likelihood of each shot, by implementing logistic regression, they found that including match context lowered the model’s average error from 0.1745 to 0.1662, indicating that it is a useful variable to add to expected goals. Shot type is therefore another very common feature discussed in the literature surrounding expected goals, included as at least one of the two variables mentioned above [2, 13, 15, 18, 20].

Various other features which might influence the likelihood of a shot being a goal were examined in Kharrat et al.’s [20] study, on the topic of ‘plus-minus’ ratings in football (a concept which addresses the challenging issue of determining the impact a specific player has on a target metric.) They included distance and angle variables, alongside rarer features such as ‘big chance’ and ‘goal value’ added by the data provider, Opta in this case, who introduced a boolean variable ‘big chance’ as a situation in which there is a good chance to score and ‘goal value’ as a measure which quantifies how important a goal would be in affecting the probability of the player’s team winning the match. This incorporated both the goal differential at the time of the shot and the amount of time remaining in the match. Kharrat et al. found that both variables were beneficial additions to their models.

Some researchers have attempted to integrate player ability when building their expected goals models. Eggels et al. [13] explored the predictability of match outcomes in football through the use of expected goals in their work. The overall ratings of the player taking the shot and the goalkeeper attempting to save it, from the EA Sports video game FIFA, were used as proxies for player ability. Whilst Eggels et al. did not examine the influence player ability had on the model they built, they did employ feature selection before training the model and neither the shot-taker’s nor the goalkeeper’s ability ratings were removed. This implies that both these variables had a positive impact on the model’s performance, and thus that player ability is a useful addition to expected goals. Research carried out by Madrero et al. [15] and Kharrat et al. [20] took a similar approach to incorporating player ability into expected goals models (i.e., through features engineered using data from the FIFA video game). They also both deemed the factor’s inclusion to have a positive impact on the performance of their models. Having found that most of the shot location and shot type features placed higher for importance, Madrero et al. did, however, point out that “being a talented player will help you score more goals, but other positional and contextual factors are more determinant” [15].

Another key area within football analytics, and one which will be addressed in this research, is match outcome prediction. Unlike expected goals, this is a topic which has been researched extensively in previous works. Undoubtedly, the most common feature included when predicting the result of a match is home advantage, a phenomenon which is prevalent in many sports. Falter and Perignon [21] incorporated home advantage, through a home/away categorical variable. All three of the models they built showed that teams were statistically more likely to win playing at home, when compared to playing away. Joseph et al. [22] also examined the effect that home advantage has on match outcome. Their paper focused on the performance of Bayesian networks (BNs) in the prediction of Tottenham Hotspur football results over the period 1995–1997. The model which gave the highest percentage of correct predictions, with 59.21%, was the only one to include a venue variable, implying in part that playing at home does have a significant impact on the match outcome. Since home advantage is a phenomenon which has been proven to occur in most sports, including rugby [23] and cricket [24], it is discussed in one form or another in many studies written on the topic of match outcome prediction [9, 25–29].

Another intuitive feature to examine when exploring match outcome prediction is the form the football club is in. Goddard [26] analysed this factor through the inclusion of a variable representing a team’s form, explicitly defined the average result (1 = win, 0.5 = draw and 0 = loss) over the most recent *n* games. Out of the 24 form variables (split evenly between the home and away team (12 each)) tested with varying values of *n*, 20 coefficients were found to be significant and in the expected sign direction (i.e. positive for the home team and negative for the away team). Baboota and Kaur [25] also addressed form in their work. They aimed to manipulate data in order to produce a feature set which could accurately predict the results of football matches during the 2014–15 and 2015–16 Premier League seasons. They engineered a variable, labelled ‘weighted streak’, which was calculated by averaging a team’s points over *k* games but, in addition, assigned greater weight to points gained from more recent matches within the period. Baboota and Kaur analysed feature importance within their best model and found that the differential between the ‘weighted streak’ values of teams facing each other was the 11th most important variable in the model. Given that their models consisted of 33 variables in total, this demonstrates that form is a relevant inclusion when predicting match outcome in football. It is for this reason form is discussed frequently in the literature [21, 27, 29].

Elo ratings can be used as a proxy for team quality in match outcome prediction. The term, coined by Arpad Elo, originates from chess and was created to rank players, with changes in the rating being scaled according to the level of opposition faced. Since its inception, it has been adapted to other sports, including football. In their work, Hvattum and Arntzen [30] compared the match outcome predictability of Elo ratings against several benchmark prediction methods, employing two loss functions (quadratic and informational) in order to evaluate the performance of each prediction method. They found that, on 15,181 matches played between 1993 and 2008 within the English league pyramid, the average values for the Elo rating’s loss functions were bettered only by two other approaches utilising odd predictions from betting sites. This is unsurprising since gambling companies tend to build much more complex models and include more variables than Elo ratings. These findings prove that Elo ratings provide some important information when modelling match outcome in football and is why similar features have been included in other works [25, 27].

The remainder of this paper is structured as follows: the Material and methods section describes the features used when modelling xG, the Results section examines the distribution of the features and discuss the findings produced from modelling expected goals across multiple leagues, and also explores the results obtained from comparing the predictive ability of traditional metrics within football against xG.

## Materials and methods

The data required to build any expectation model in football (event and/or positional information) is hard to obtain as the companies who collect the data usually use it to build their own models. Fortunately, as part of the Soccer Data Challenge initiative (a football analytics event held in Italy [16]) the organisers provided what they believe to be the largest collection of event data ever released to the public. The data, which was collected by Wyscout (another leading football analytics company), comprises all match events from the top 5 leagues’ (English *Premier League*, Spanish *La Liga*, German *Bundesliga*, Italian *Serie A* and French *Ligue 1*) 2017–18 seasons. Despite the fact that there is no positional data (so some influential features examined in other works [2] cannot be included in the models), this dataset was the most complete, publicly-available source that was found, and crucially, contains the necessary information required to fulfill the objectives of this study.

### Wyscout data

Event and match data for each league as well as information on all the players, teams, PlayeRank [31] values, competitions, coaches and referees is contained in the Wyscout dataset. The following common xG modelling features were manipulated using these datasets:

- **Distance**: the Euclidean distance from the coordinates of the shot (*x*, *y*) to the centre of the goal.
- **Angle**: the angle the location of the shot makes with the centre of the goal.
- **Body Part**: this includes head/body, strong foot and weak foot.
- **Match Situation**: this includes open play, counter, free kick and penalty. If any information relating to the latter three values was absent, the shot was assumed to be from open play.

The following rarer xG modelling features were also available in the Wyscout data. Since these variables are either not common or previously untested, a brief description on the motivation behind their inclusion is given.

- **Side**: whether club involved was playing at home or away. Home advantage can play an influential role in the match outcome. This feature was therefore included to determine whether this phenomenon exists in an intra-match setting.
- **Position**: the general position the players plays on the pitch, taking the values defender, midfielder or forward. Since shot-taking ranks high amongst the roles of some positions on the field (e.g., forwards) and ranks low amongst others (e.g., defenders), it is natural to assume this feature is influential.
- **Gameweek**: which gameweek the match was played in. This variable was included to examine whether goal probability could differ depending on the period of the season the game is played in (e.g., it may be lower earlier on in the season due to lack of focus and may be higher later on in the season due to greater pressure to score).
- **Time of shot**: the time (in seconds) the given shot occurred in the match. Similar to the gameweek variable, this feature was included to determine whether time was a factor in goal probability.
- **Goal Difference**: the number of goals the shot-taker’s team is leading or losing by at the time of shot. This feature examines whether players may be more likely to score if they are leading in a match (since they could be more relaxed with the knowledge that scoring a goal is not a necessity) or more likely to score if they are losing.
- **Length of Possession**: the length of time (in seconds) that the team had been in possession of the ball before the shot occurred. This feature was included to investigate whether getting the ball into a shooting position quickly or more slowly might affect the probability of a goal.
- **Age**: the age of the shot-taker on the date of the match. This variable explores whether experience can influence the likelihood of scoring a goal.
- **Current Rank**: the position the shot-taker’s team occupies in the league table on the date of the match.
- **Previous Season Ranking**: the position the shot-taker’s team placed at the end of the previous season. Due to the relegation/promotion system, some teams did not have a previous season ranking. Teams that had been promoted were assigned the previous season ranking of the teams that had been relegated the season before, according to the order they were promoted.This was included to examine the effect of team quality on goal likelihood.

In addition to the Wyscout data, information from Fbref was sourced in order to create the variable **Match Attendance**. FBref [32] is a site which provides football statistics and history for over 100 men’s and women’s club and national team competitions, with data freely shared in csv format. The reasoning behind the integration the Match Attendance variable is that higher figures could create an atmosphere in which all actions are more pressurised, this possibly influencing goal probability of a given shot. Fbref also share data on the expected goals values in games, calculated by StatsBomb, a leading football analytics firm and competitor to Wyscout. This information will be used when comparing the predictive ability of expected goals against traditional metrics, examined later on.

A variety of more complex features were further added at the xG modelling stage. These will be discussed separately in the following subsections.

### PlayeRank

Wyscout data includes details on match PlayeRank scores for players. This metric, first developed by Pappalardo et al. [31], aims to assess player performance. Whilst expected goals can aid player performance evaluation, due to the fact that it is based around shots, it is not so easily applied to defenders and some midfielders, whose roles usually do not involve shot-taking. This is why a statistic which can be assigned to all outfield players is valuable (goalkeepers require a separate analysis [31]).

To address this issue of complexity, PlayeRank follows a procedure that consists of three phases: rating, ranking and learning. The rating phase is split into two parts—individual performance extraction and player rating. Individual performance extraction concerns the building of a 76-dimensional feature vector for each player in each match. During the player rating stage, the scalar product between this vector and the feature weights (computed as part of the learning phase) is calculated and then this figure is then normalised so the resulting value is between 0 and 1. The process is defined in [31] as:

$$\begin{array}{c} r\left(u,m\right)=\frac{1}{R}\sum_{i=1}^{76}w_{i}x_{i} \end{array}$$

where *r*(*u*, *m*) is the base PlayeRank value for player *u* and match *m*, *R* is a normalisation constant, *w*_*i*_ are the feature weights and *x*_*i*_ are the feature values. The ranking phase involves applying a *role detector*—an algorithm, trained during the learning phase which assigns a player to one or more roles based on their average position on the pitch during a match. This helps to produce a set of role-based rankings. A player is then categorised into one of the roles if they have at least 40% of the matches assigned to that role, a value Pappalardo et al. found to optimal after testing for different percentages. Finally, the learning phase consists of two sections—role detector training and feature weighting. Role detection applys a *k*-means clustering algorithm (with hyperparameter *k* set to 8) on the average *x*, *y* coordinates the player has over a specific match.

For each player and before each match, PlayeRank values from all previous matches the player was involved in were summed to produce the cumulative PlayeRank score variable. This feature was included in order to account for player ability, with the assumption that players with frequently high PlayeRank scores usually have a higher chance of scoring a goal when taking a shot.

### Match importance

The importance of a match is difficult to quantify and depends on factors including the location and history of both clubs, past results between the teams and where they are placed in the table on the day of the match. An attempt to create a statistic for match significance was first attempted by [33] for sport Australian Rules Football and later adapted for association football by [34].

The process for calculating match importance assigns a reward to each position in a league, e.g. qualification for the UEFA champions league involves the first 3 places of the Premier League and La Liga, definite survival is the position immediate above the the highest ranked team in danger of relegation.

Before each gameweek, the expected number of points required to finish the season in each of the corresponding positions, given the number of points attained up to that gameweek, is then computed. We do this by following the approach in [34] which takes the number of points the team occupying the position in question in Table 1 has before the gameweek in question and multiplying it by inverse of the proportion of season that has been played so far, then subtracting the number of points a team has at that time. We take into account scenarios in which it is deemed impossible for the team to finish the season lower than the designated position, by taking the maximum value between this figure and 0.

$$\begin{array}{c} RP_{i}\left(g\right)=max([\frac{TP_{1st}\left(g\right)\times M}{g}]-TP_{i}\left(g\right),0) \end{array}$$

where *RP*_*i*_ is the required points for the team in position *i*, *TP*_*i*_ is the number of points the team in position *i* has, *g* is the number of gameweeks played so far in the season, and *M* is the number of matches each team plays in their league season.

**Table 1:** League positions resulting in specific consequences for teams in each league.

	Premier League	La Liga	Bundesliga	Serie A	Ligue 1
Champions	1st	1st	1st	1st	1st
Automatic UCL Qual.	3rd	4th	4th	4th	3rd
UCL Play-Off	4th	-	-	-	-
Automatic UEL Qual.	6th	7th	6th	7th	6th
UEL Play-Off	7th	-	-	-	-
Definite Survival	17th	17th	16th	17th	17th
Possible Survival	-	-	17th	-	18th

Next, for each position, the probabilities that a team will earn the required points within the remainder of the season, given that they win or lose their upcoming match are sampled from the cumulative binomial distribution function. This function computes the likelihood that a given number of successes will be observed out of a given number of trials, based on a given probability of success.

For use creating the match importance feature in football, the number of trials is given as the maximum possible points available in the remainder of the season and the no. of successes is given as the required points for the team in question (as computed above). The probability of success is chosen to be 0.5 since Bedford and Schembri find it to be the optimal value in their study.

Match importance was then defined as the difference between these two probabilities. In this way, it represents the significance to the team of winning their next match given their position in the table.

This feature was included to assess the influence that psychological pressure can have on players when shooting. Players may tend to perform better, or possibly worse, if their team’s future success rides heavily on the outcome of the match.

### Team form

Team form has been addressed in multiple papers on the topic of football match outcome. One method is to assign 1 to a win, 0.5 to a draw, 0 to a loss, and then take the average of these values over a predetermined set of matches [26]. The approach taken in this study originates from a previous work on modelling football results in the Premier League [25].

A team’s form before the *j*th match, *ω*_*j*_, is defined in [25] as a weighted version of team form by

$$\begin{array}{c} \omega_{j}=\sum_{p=j-k}^{j-1}2\frac{\left(p-\left(j-k-1\right)\right)r_{p}}{3k(k+1)} \end{array}$$

where *k* is the number of matches included in the form variable and *r*_*p*_ is the result (3 for win, 1 for draw, 0 for loss) of the *p*th match in the sum.

The numerator, (*p* − (*j* − *k* − 1))*r*_*p*_, expresses how many points the team has gained within the given window *k*, with each points value weighted from 1 to *k*. That is, for the first match in the window examined, *p* − (*j* − *k* − 1) = 1, for the second, *p* − (*j* − *k* − 1) = 2, and so on, up until *k*. The denominator, $\frac{3k(k+1)}{2}$, expresses the maximum number of weighted points a team can attain within the given window with *k* = 6 found to produce the best predictions [25].

This feature included to assess differences in team quality when modelling expected goals, the better a team has been performing over recent matches, possibly the more likely they will be clinical in their next match.

### Elo rating

The Elo metric, first proposed by Hungarian-American physics professor Arpad Elo for use in chess, aims to evaluate player/team skill by taking into account previous rating and match results. The original methodology has been adapted to football [30].

Elo ratings were taken from Clubelo [35]. Each team’s Elo rating is modified after each game according to

$$\begin{array}{c} \Delta_{i}=k\left(r-P_{i}\right) \end{array}$$

where *r* is a quantified version of team *i*’s result against team *j* (1 for a win, 0.5 for a draw and 0 for a loss) and *k* is a hyperparameter which can be tuned to determine the scale at which the teams’ ratings are altered (the higher the value of *k*, the more weight each result has on teams’ future scores), here *k* = 20 [30]. *P*_*i*_ is team *i*’s pre-match win probability

$$\begin{array}{c} P_{i}=\frac{1}{1+10^{\frac{\beta_{j}-\beta_{i}}{400}}} \end{array}$$

and *β*_*i*_, *β*_*j*_ is team’s,opposition’s pre-match Elo rating.

If a higher-quality team (i.e., one with a much larger Elo rating) faces a lower-quality team and the former defeats the latter (the expected score), both the former’s reward and the latter’s punishment are not overestimated.

This feature was included in order to incorporate team quality into the expected goals models. It is hypothesised that the higher a team’s Elo score, the more likely a shot from one of their players will result in a goal.

Elo as a metric has some important shortcomings though, it may be inflated by beating the same opponent on several occasions as may happen over several seasons in a football league and may pose a problem when used in optimisation models, see [36] for an interesting discussion. In this research, we restrict the dataset to a limited number of seasons to avoid much of these issues.

### Player value and average transfer spend

Player transfer values were obtained from Transfermarkt [37], a site which specialises in football player transfers with details on general news, rumours and player market value. This dataset was joined to the Wyscout dataset using the python library, fuzzymatcher [38], which allows datasets to be combined without common identifiers, based on one or more shared fields.

The Transfermarkt and Wyscout datasets were merged on player name, team, birth year, height, position and strong foot. Matches with equivalent birth year and a score above 5 were assumed to be correct. All other matches were inspected to either verify or reject the prediction made. Rejected matches were replaced with the correct player value by manually inputting the data from Transfermarkt directly.

Transfermarkt estimate players’ values based on a multitude of factors, ranging from their age and reputation of the league the play in to their susceptibility to injuries and market demand. Figures for player values can additionally be altered by any transfer fees they are involved in and the circumstances surrounding said transfer. This feature was included at the modelling stage with the aim of incorporating player ability, since, naturally, the better the player, the more valuable they are.

A team’s average transfer spend was calculated by simply summing the cost of each incoming transfer (including loan fees) in the summer window (i.e., before the season starts) and dividing this total by the number of transfers the club made during the same window. The motivation behind the inclusion of this factor is to integrate team quality into models, due to the reasoning that the teams with better players tend to sign more valuable players.

### Modelling

The aim of this section is to describe the machine learning models used. It is not a primer on machine learning, for explanations of machine learning and boosting algorithms see, for example, [39–42]. For all models assessed, a standard train and test data split of 70% to 30% was chosen. Since goals are relatively rare (with roughly one goal scored every tenth shot taken), training and test data were stratified for expected goals modelling so that the proportions of goals were equivalent in both datasets. We scaled the features in the model to avoid larger weights being assigned to variables with larger values and vice versa, regardless of the feature’s true impact on the model’s output. The values of features are altered according to (Min-max scaling)

$$\begin{array}{c} x^{\prime }=\frac{x-\text{min}(x)}{\text{max}(x)-\text{min}(x)} \end{array}$$

We used cross validation with 10 folds to examine how our model captured trends in the data. *k*-fold cross validation is a process in which the training dataset is first split into *k* sample groups (or ‘folds’). Next, the model is trained on *k* − 1 folds and evaluated on the remaining one. This action is repeated for each of the folds created, giving *k* scores which are then averaged to produce an overall value.

For each of the algorithms applied to expected goals modelling, hyperparameter tuning is carried out through a grid search to perform better on the data provided. For a particular model, various values of the hyperparameters are chosen and an exhaustive search of all combinations of the entries provided is executed. At each of these combinations, the algorithm is trained and 10-fold cross validation is used to evaluate its performance. Once all of these combinations have been searched through, the one which produced the optimal evaluation score is chosen as the tuned model.

For classification algorithms, the standard cost function used is log loss. For models with binary outputs, such as is the case with expected goals models, the log loss function is given by [43]

$$\begin{array}{c} l=-\frac{1}{n}\sum_{i=1}^{n}y_{i}\text{log}\left(p_{i}\right)+(1-y_{i})\text{log}(1-p_{i}) \end{array}$$

where *n* is the number of data points, *y*_*i*_ is the *i*^th^ numerical value of the dependent variable (0 if negative outcome, 1 if positive) and *p*_*i*_ is the probability of a positive outcome from the *i*^th^ value of the dependent variable. This function is minimised during the training process.

For multi-class classification problems this is simplified to

$$\begin{array}{c} l=-\frac{1}{n}\sum_{i=1}^{n}\sum_{j=1}^{m}y_{ij}\text{log}\left(p_{ij}\right) \end{array}$$

where *m* is the number of classes, *y*_*ij*_ is the binary value of the *j*^th^ class (i.e. 1 if it is a member of the class, 0 if it is not) and *p*_*ij*_ is the probability that the *i*^th^ data point belongs to the *j*^th^ class.

Another cost function used in this research is the Gini index [39]. The Gini Index gives an idea of how varied a resulting node is by calculating the density of each class in the sample produced from the split.

$$\begin{array}{c} G=\sum_{k=1}^{m}p_{k}(1-p_{k}) \end{array}$$

where *m* is the number of classes in the output variable and *p*_*k*_ is the proportion of values in the *k*^th^ class out of the number of data points left in a given split.

We evaluate each machine learning algorithm after the training phase in order to decide on which model to use. Whilst most evaluation metrics for classification problems involve analysis of the model’s predictive ability in various situations (e.g. predicting negative outcomes accurately), this approach does not suit expected goals modelling. This is because the only result from the model which is of use is the probability that a specific shot is part of the ‘goal’ class, and not a prediction of whether a shot is a goal (i.e. a binary output). Here we used the log loss, the lower this score, the better the classification algorithm is at accurately estimating goal probability.

We compared the following machine learning algorithms when building our expected goals model:

**Multi-Layer Perceptron (MLP)** We used the logistic equation as the MLP’s activation function [44] and using the log-loss as the cost function. The hyperparameters and their values, included as entries into the grid search algorithm to help fine-tune the MLP are:

- Solver *lbfgs*, *sgd* and *adam*: This hyperparameter decides on which approach to changing the MLP’s weights is taken
- Hidden Layer Sizes—100, 250 and 500: the number of nodes included in each hidden layer of the MLP.
- Alpha (*α*)—0.0001, 0.01, 0.1, 1, 10: The parameter which decides the influence the penalty argument has on the model.
- Maximum Iterations -100,300,500: how many times before the model stops performing the back propagation.

Boosting algorithms attempt to strengthen the performance of weak learners to create a strong learner by iteratively fitting a new weak learner (in this study, decision trees) based on the predictions that were made by the previous one. In this study, two boosting algorithms have been chosen to model expected goals—AdaBoost and XGBoost.

**AdaBoost**(short for ‘adaptive boosting’) builds a collection of what are termed ‘stumps’ at each iteration. The following hyperparameters and their values were included in the grid search algorithm:

- Number of estimators 25, 50, 100 and 200: This decides how many stumps are added to the AdaBoost’s forest.
- Learning rate—0.01, 0.1, 1, 10, 100: value which affects how large stump’s error rate becomes at each iteration.
- Algorithm—*SAMME*, *SAMME.R*: different approaches to updating the weight values of data points.

**XgBoost** (short for ‘extreme gradient boosting’) takes a different approach to ensembles of weak learners, by employing gradient descent methods. At each iteration of the algorithm, XGBoost builds decision trees (with a depth specified before training the model) using the residual errors of predictions made by the previous tree. The values of the hyperparameters used to fine-tune the performance of XGBoost were:

- Eta (*η*)—0.01, 0.1, 0.3 and 0.5: The learning rate for the calculation of updated probabilities.
- Objective—*binary:logistic*, *binary:logitraw* and *binary:hinge*: the method for producing probability estimates.
- Maximum Depth—3, 5, 7 and 10: the depth of each decision tree built, with higher values creating more complex trees (and therefore possibly leading to over-fitting).

For AdaBoost, the normalised total decrease in the Gini Index score generated by a feature is taken as the feature’s influence on the model’s output. XGBoost includes several metrics to assign feature importance to its input variables. Gain, which is the most relevant measure to indicate relative feature importance in a model, is the improvement in predictability attained by the variable to the splits it makes. The reasoning behind the metric is that adding a certain split from the variable in question led to some wrongly classified outputs being correctly categorised.

## Results

Before building expected goals models using these features, it is important to examine their distributions in order to both observe whether the theorised effect they have on goal probability is valid in practice and gauge the extent of their influence.

### Distance and angle

**Fig 1:**
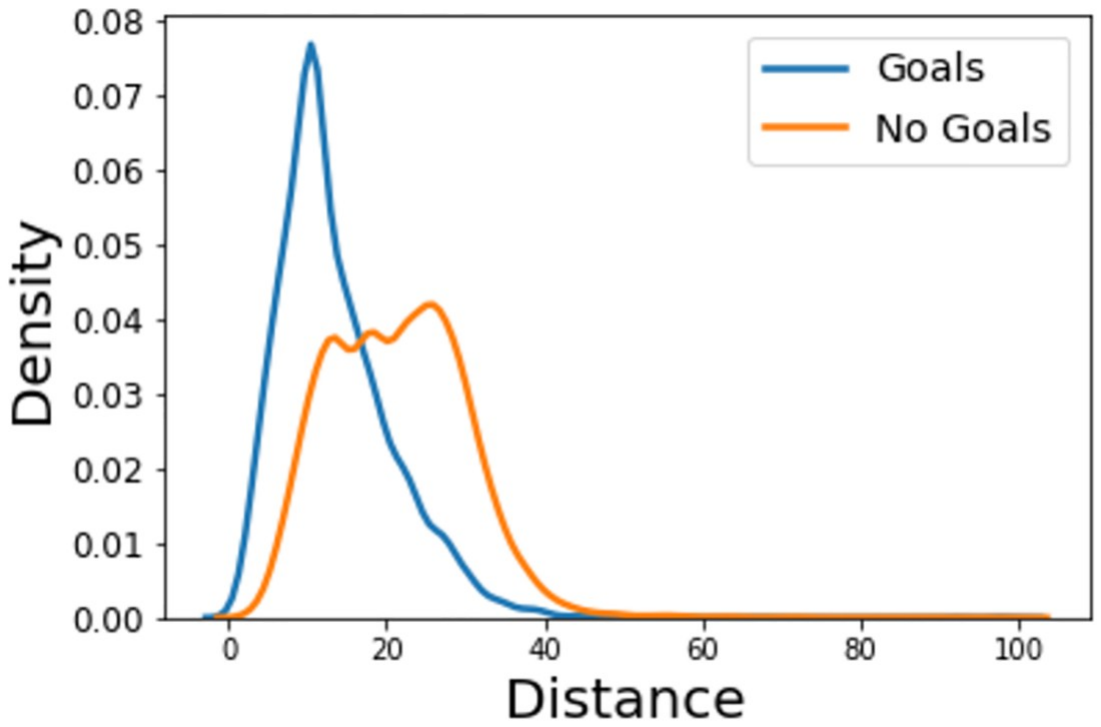
Kernel density estimate of the distance a shot is taken from for those that result in a goal or miss/save.

**Fig 2:**
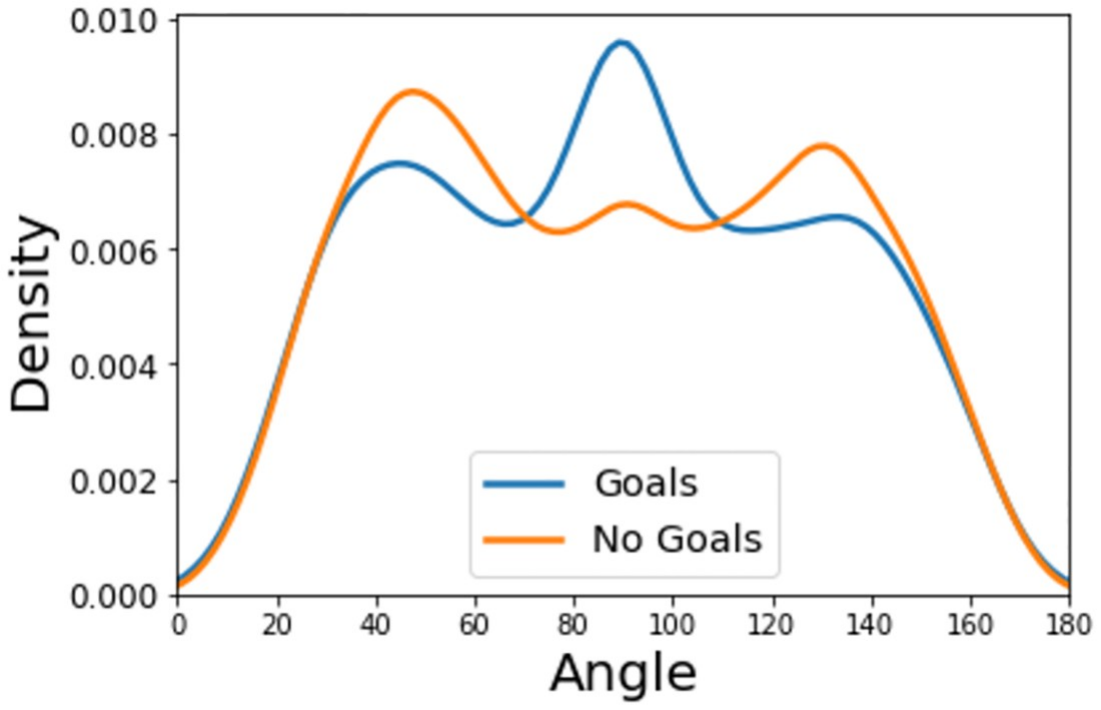
Kernel density estimate of the angle a shot is taken from for those that result in a goal or miss/save.

The two most common features included in expected goals models are distance and angle. Figs 1 and 2 show the distance and angle that result in a goal or no-goal.

What is immediately noticeable is that, whilst many shots are taken from a relatively long distance, only the much closer attempts tend to result in a goal with goals become less likely to occur than saved and off-target shots at a distance of around 20 metres. When comparing this with the hexbin plots, it is clear that many shots which do not result in a goal are taken within this range (>20m). This is just one of the many reasons why expected goals models are so useful for managers and coaches.

**Fig 3:**
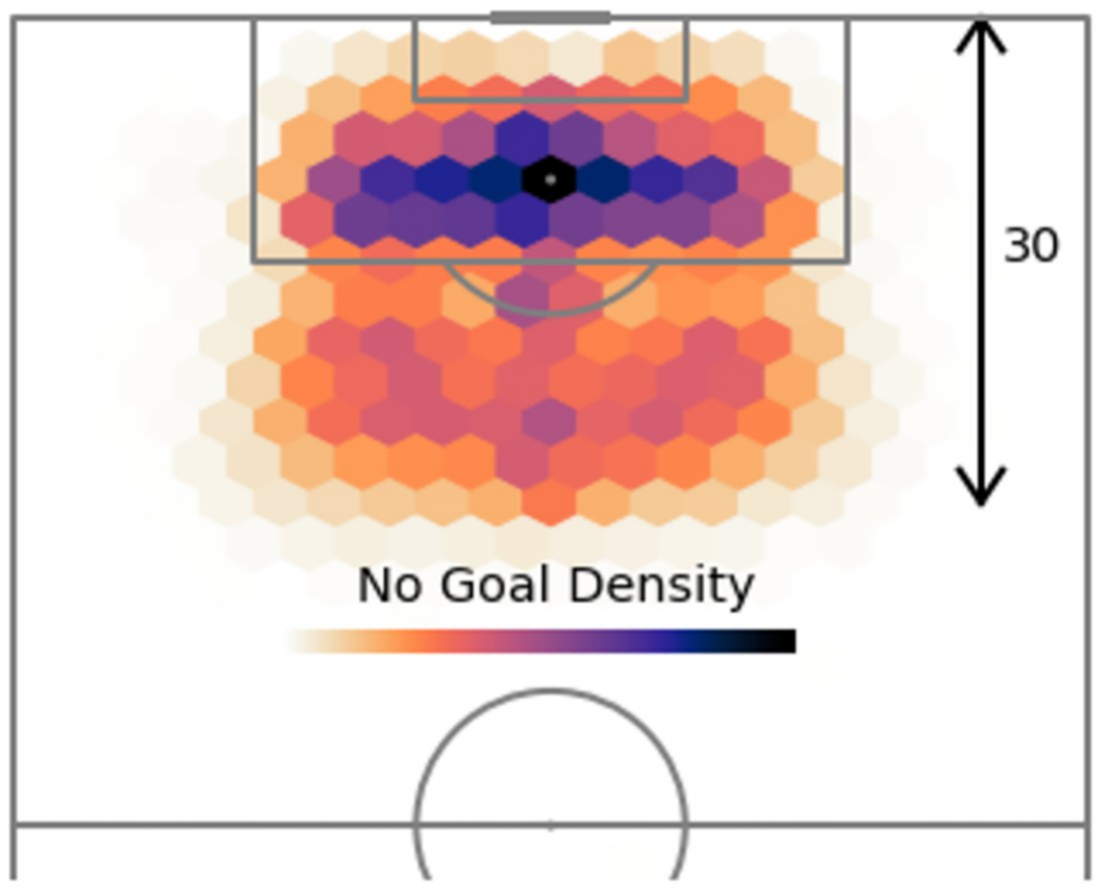
Heatmap of the where shots are taken from that do no result in a goal.

**Fig 4:**
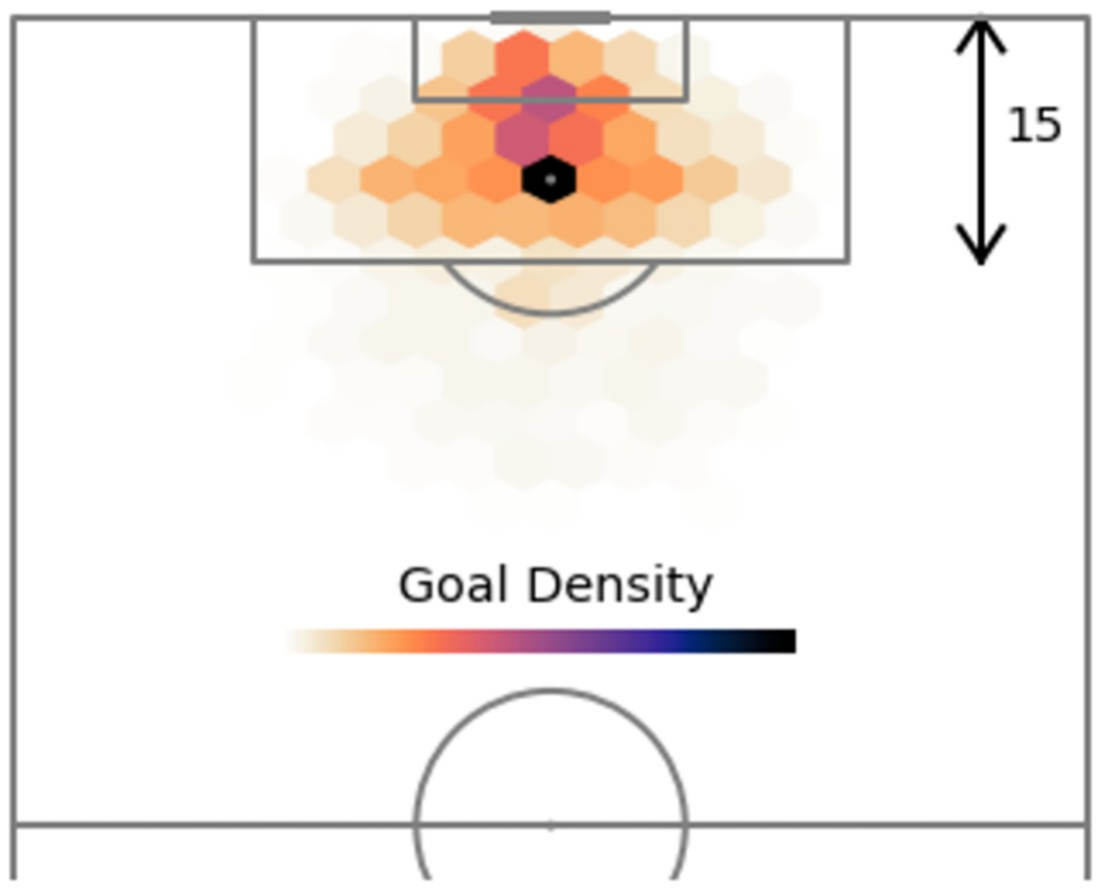
Heatmap of the location of successful shots.

The hexbin plots also reveal information about the influence angle has on shot outcome. Figs 3 and 4 show that the majority of unsuccessful shots occur within the range ∼40° to ∼140°, whilst successful shots (i.e., goals) tend to appear within a narrower set of values (between ∼60° to ∼120°). This is also evident from inspection of the angle kernel density curves, with three visible peaks, two of which more likely to contain unsuccessful shots.

One other prominent aspect of the angle feature, exhibited in Figs 2 and 4, is that shots tend to originate more from positions slightly left to the centre, than right of the centre. This phenomenon implies that there are three scenarios attempts on goal usually occur in: one in the middle of the pitch and two taken just either side of the centre in order to create a better angle to shoot from, usually with a player’s strong foot. Since right-footed players are more common than left-footed players, this explains the slight difference in shot/goal density between both these sides.

### Time of shot

**Fig 5:**
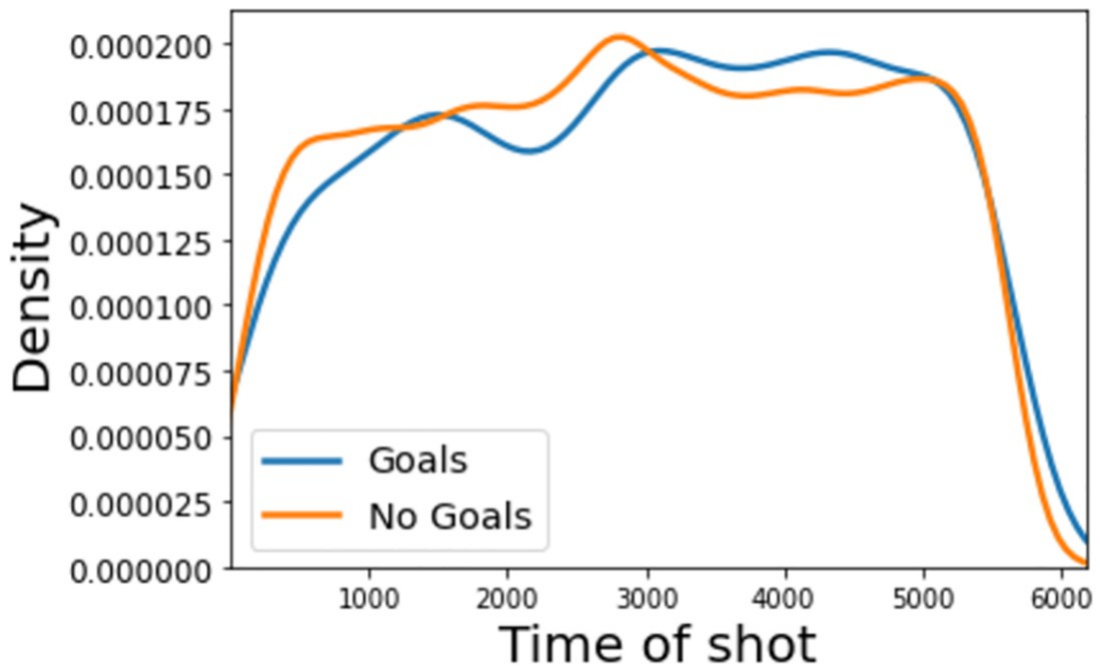
Kernel density estimate for the time a shot is taken (in seconds from the start of the match).

The kernel density plot for the shot time feature, Fig 5, indicates that shots which occur later on in matches have a slightly higher chance of being successful. More specifically, teams tend to not start games with high levels of shot proficiency, with shots more likely to be unsuccessful than successful in the first 20 minutes. This does change briefly midway through the first half, suggesting that teams have greater knowledge of how the opposition wants to play and have therefore found ways to create clearer goal-scoring opportunities. It then immediately dips before half-time (∼2700 seconds), maybe because teams recognise the value in maintaining a scoreline until half-time, when they can reevaluate and gain feedback, instead of risking losing their lead or conceding more if they increase attacking efforts, thus weakening their defensive structure. After half-time, perhaps as teams have changed tactically, or simply as both teams take greater risks with less time left to affect the scoreline, the likelihood of a shot resulting in a goal increases significantly to where there is more chance of a shot being goal than it being saved, off-target or blocked.

### Player value

**Fig 6:**
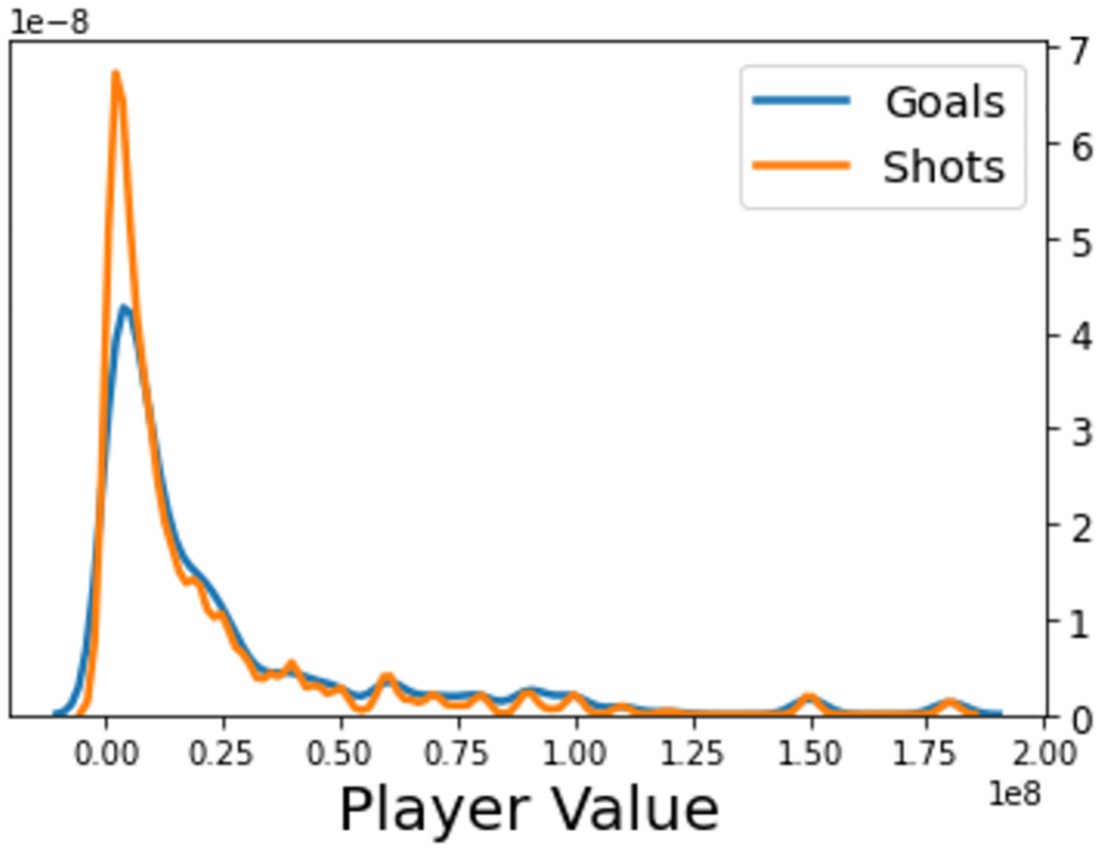
Kernel density estimate of the player value.

The majority of each player’s Transfermarkt evaluation, Fig 6, are close to zero, with few players reaching the highest values. This matches with what is expected for a distribution representing player ability. Most players produce low values and those that do not become members of an exclusive minority. Disparities between goal and unsuccessful shot densities also reflect this distinct separation. Players with low values have almost a 2 to 1 ratio (saved/off-target shots to goals), whilst the higher-valued footballers achieve a ratio closer to 1 to 1. When compared with the other variables featured in this exploratory analysis, these trends much less prominent, perhaps indicating that player value may not be deemed an influential inclusion in the expected goals models built in this paper. Although, these findings could simply be due to the fact that, whilst player position will be accounted for when estimating xG values, the kernel density plot does not take into consideration this dependence. After all, it is logical to imagine that, in general, a more expensive player is better at fulfilling their role within the team than a cheaper alternative. However, with different roles comprising sometimes vastly different responsibilities, it is unnatural to presume that value and shot proficiency are positively correlated for all players.

### Elo rating

**Fig 7:**
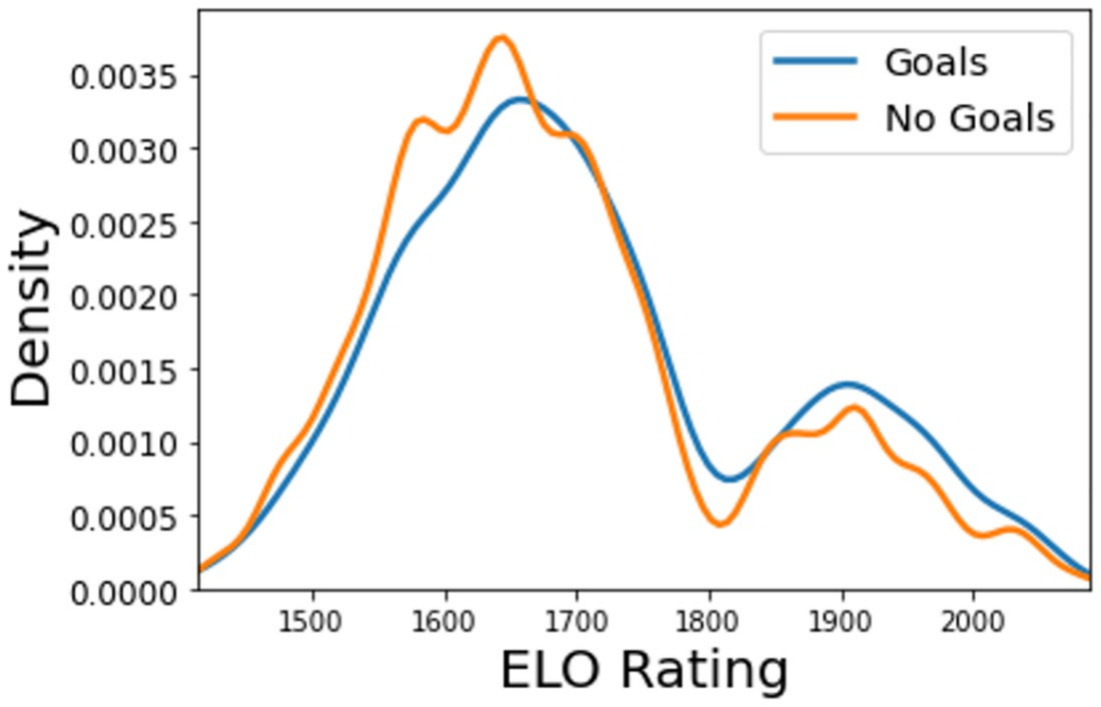
Kernel density estimate of the ELO rating.

From the Elo ranking, Fig 7, it is clear from its distribution that the better the team’s ranking, the more likely they are to score a goal from a given shot. Teams with a relatively low score (∼1450−1650) tend to be most frequently unsuccessful with their shooting. For mid-range teams (∼1650−1850) in the dataset, this trend changes. This gap then widens for the highest rated teams (∼1850−2050).

One additional interesting conclusion that can be drawn from this plot is that, much like with player value, there appears to be a distinct separation (in terms of Elo ratings) between most teams and a smaller group of elite teams, due to the two prominent peaks in the graph. This split is emphasised by the fact that, out of all of the five seasons prior to the 2017/18 season (which is examined in this study), in all of the top five leagues (English, Spanish, German, Italian and French), only one team to win their league does not appear in this upper Elo rating range—Leicester City (*Premier League* champions in 2015/16).

#### Expected goals modelling results

A set of 20 features, detailed in the Materials and methods section, was engineered for each of the top five football leagues (English *Premier League*, Spanish *La Liga*, German *Bundesliga*, Italian *Serie A* and French *Ligue 1*) and one for all leagues combined, using data from the 2017–18 season. In order to prepare these features for modelling, categorical variables were either encoded 0 and 1 for values if binary or one-hot encoded if non-binary. Variables were then min-max scaled and a training and test data split of 70% to 30% was applied, with stratification to ensure that both groups contained a similar ratio of positive to negative outcomes (i.e. goals to unsuccessful shots). For each of these leagues, these features were used as inputs into machine learning and statistical models; logistic regression, multi-layer perceptron (MLP), random forest, AdaBoost and XGBoost. Unlike conventional classification problems, where the target variable is binary (i.e., a prediction of 0 or 1), expected goals models output probabilities that a specific shot is a goal. Hence, the metric employed for evaluation of the xG models built was chosen to be log loss. Naturally, the lower this score, the better the classification algorithm is at accurately estimating goal probability. 10-fold cross validation was used to determine each model’s performance on the training sets, with log loss scores calculated on test sets only after these values were analysed to verify whether alterations to the models needed to be made, each model was further tuned by selecting a variety of its algorithm’s hyperparameters and executing an exhaustive search over each combination of their chosen values (i.e. a grid search approach). Once this was complete, the combination which produced the optimal evaluation score became the algorithm’s tuned model. The above procedure was then repeated for this model, thus producing training and test log loss scores, both before and after hyperparameter tuning, for each classification algorithm.

#### Test data log loss scores

Since test scores help to decide which model generally performs best, these are the values shown in Table 2.

**Table 2:** Log loss test set scores for each league and model, before and after tuning (LR = logisitic regression, RF = random forest, AB = AdaBoost, XGB = XGBoost). The best score for each league is highlighted in bold.

League	Before Tuning					After Tuning				
	LR	MLP	RF	AB	XGB	LR	MLP	RF	AB	XGB
Premier Leaguex	0.28554	**0.28315**	0.36957	0.66474	0.38324	0.28364	0.28337	0.30365	0.31471	0.29268
La Liga	**0.30629**	0.31796	0.34123	0.66277	0.41489	0.32109	0.31975	0.31128	0.32538	0.31397
Bundesliga	0.29629	0.28814	0.33268	0.66909	0.34123	0.28685	0.2883	0.29733	0.31481	**0.28425**
Serie A	0.28907	0.2841	0.29934	0.67201	0.32746	0.28945	0.28922	0.29233	0.30801	**0.28295**
Ligue 1	**0.29118**	0.29171	0.34387	0.66371	0.36942	0.29366	0.29873	0.30114	0.32408	0.29752
All Leagues	0.28614	0.285	0.30698	0.671	0.30594	0.28563	0.28286	0.2897	0.31368	**0.28184**

In general, most of the models (with the exception of AdaBoost) performed relatively well on data from the *Premier League*. Surprisingly, despite the complexity of artificial neural networks and thus greater ability to capture significant trends in the data, this was the only league for which the MLP produced the best results. Tuning the *Premier League*’s optimal model further actually led to a decrease in predictability, indicating that this updated model suffered from over-fitting.

Values from *La Liga* data indicate that capturing trends which affect shot outcome, and thus explaining the randomness inherent within goals, was difficult to achieve for the Spanish league. Results for this competition tended to be worse than other, with none of its test data scores below 0.3. This was not the case for the rest of the top five leagues (and all leagues), with 3 of the 5 algorithms assessed producing optimal values lower than this number.

What is first noticeable from *Bundesliga* results is that most models produced very competitive scores after tuning hyperparameters (when omitting AdaBoost), with the remaining four algorithms yielding scores all within 0.01308 of each other. The scores generated are slightly more impressive when considering that the event dataset for the *Bundesliga* was the smallest of any leagues, due to the fact that fewer teams are involved in the competition.

For *Serie A* data, all algorithms produced very low optimal scores on test data, with most generating values below 0.3. Moreover, the AdaBoost models do not give better scores on the data for any other league, despite it not surpassing the 0.3 threshold. Strangely, whilst all ensemble methods show strong results (relative to other leagues), both the logistic regression and MLP algorithms perform poorly when making the same comparison. This somewhat strays from the norm, indicating that decision tree-based models were able to capture the trends within *Serie A* with greater ease than their counterparts.

Findings from modelling *Ligue 1* data show that the features engineered as part of the analysis into expected goals are reasonably adept at explaining much of the randomness within goal probability for the French league. Most values (apart from those for AdaBoost and random forest) are consistent for both training and test scores after tuning, with a lowest value of 0.28456 and highest of 0.29873. The non-tuned logistic regression model is once again shown to be the best performing algorithm (similar to *La Liga*).

Finally, scores from data on all leagues combined are the most impressive out of all the leagues’ optimal models. Results from each of three best performing models are at most 0.28563 for logistic regression, a significantly strong value in of itself, and 0.28184 for XGBoost. These findings included the only set of values within which each model produced a better log loss score after alterations were made to their configuration. This is most likely due to the wealth of data the all leagues models had as inputs, meaning that trends were easier to capture for each algorithm, when compared to the other optimal models built.

Differences between results for each league’s AdaBoost models before and after tuning were the largest for all algorithms assessed. Each competition’s log loss values for AdaBoost decreased from around 0.66 to around 0.31. These differences were due to both reducing the number of stumps added to AdaBoost’s forest and lowering the extent to which weights for correctly and incorrectly classified data points are altered. Most interestingly, however, is the effect of these changes on the importance of the features within the model. For each league, before tuning of the model, the AdaBoost algorithm used a mixture of features in order to make its predictions. However, after the model has been tuned, the only feature the AdaBoost took into account when making predictions was distance. The fact that these changes resulted in the AdaBoost models more than halving their log loss scores indicates just how influential distance is when developing expected goals models.

In order to gauge how impressive these results are, the best performing model from this study (tuned XGBoost for all leagues data combined) was compared to the optimal models built in various other papers on the topic of expected goals and summarised in Table 3. Since some of these studies employ other techniques to evaluate model performance, calculations were carried out on the predictions from the all leagues model to produce equivalent metrics. These included the Brier score and AUC ROC. Noordman’s [5] study into improving match outcome prediction using in-game information involved the development of an expected goals model. The optimal model built, which included data on players’ FIFA ratings as a proxy for player ability, produced a Brier score of 0.0799. When using the optimal model in this paper to predict goal probabilities from test data, it gave a superior score of 0.07908. This being said, Noordman did achieve a better log loss value (0.2787 vs. 0.28184). Since log loss punishes poor predictions more strongly than the Brier score does, this indicates that the optimal model built in Noordman’s study was slightly more consistent with its predictions. However, in Noordman’s study, when the model’s prediction for a given data point was close to the true value, the prediction output from the optimal model in this study tended to be closer. Additionally, works by both Eggels [13] and Anzer and Bauer [2] involved the formulation of different expected goals models, in part evaluated by AUC ROC. The optimal model built in both papers produced AUC ROC scores of 0.823 and 0.814, respectively. The AUC ROC score on test data for the optimal model in this study was 0.8. Whilst this result is obviously marginally worse than those reported by Noordman, both Eggels, and Anzer and Bauer used positional data, in addition to event data, in their models. This means that some influential features, which were incorporated into the research carried out by these authors, were not able to be engineered for use in this study (since, as described in Materials and methods section, the necessary data was not available). Thus, within this context, the results reported above can be deemed impressive.

**Table 3:** Summary of the results of our model compared to published models. The AUC ROC for the optimal model in this research used test data, and used players’ FIFA ratings as a proxy for player ability.

Model	Brier score	AUC ROC	Log-loss value
This model	0.0799	0.8	0.28184
Noordman [5]	0.0799		0.2787
Eggels [13]		0.823	
Anzer and Bauer [2]		0.814	

#### Feature importance

**Fig 8:**
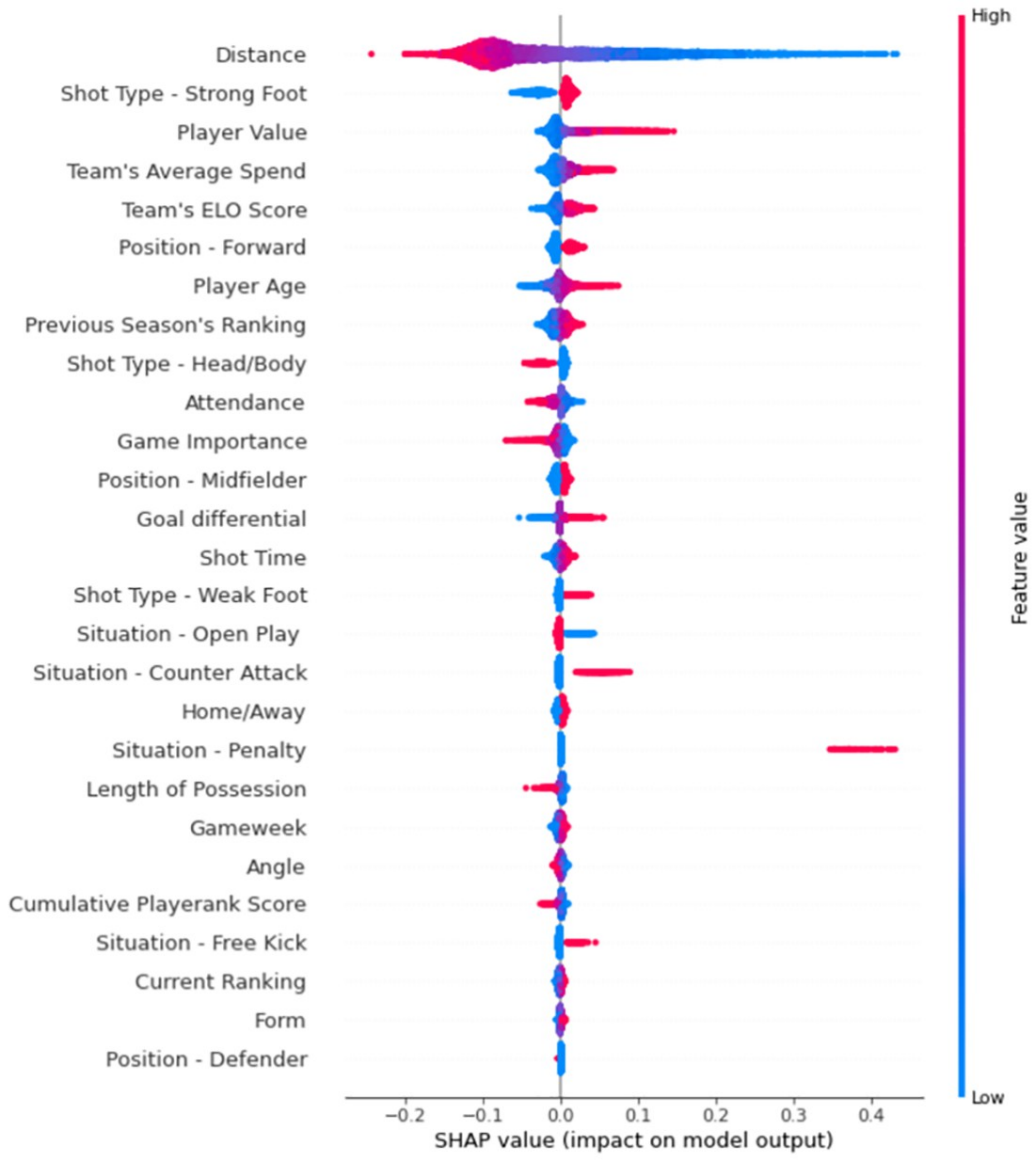
Important features for premier league, ordered by importance. In general, most of the models (with the exception of AdaBoost) performed relatively well on both training and test data, however, the MLP produced the best results on unseen data.

One of the primary aims of this paper is to analyse the influence that previously untested features could have on improving xG performance. Whilst this influence is somewhat evident in the positive results discussed above, these findings neither show which features were most and least important in making predictions, nor do they reveal how these new additions vary in impact within each of the top five leagues. It is for these reasons that a measure quantifying feature importance was computed for each classification algorithm tested. For the majority of the models built, this was a simple task. Both the size of coefficients and odds ratios were chosen for logistic regression, the former to simply compare influence and the latter to examine how different values within each feature impacted model outputs. For random forest and AdaBoost, the normalised total decrease in the Gini Index score generated by the feature in question was chosen. The Gini Index gives an idea of how varied resulting node is by calculating the density of each class in the sample produced from the split. Gain was selected as XGBoost’s feature importance measure. This is the improvement in predictability attained by the variable to the splits it makes. The reasoning behind the metric is that adding a certain split from the variable in question led to some wrongly classified outputs being correctly categorised. However, due to the fact that neural networks are so-called ‘black boxes’, it is impossible (through any value the model outputs) to explain how the network makes its predictions. Thus, Shapley values [45] (shortened to SHAP from SHapley Additive exPlanations) were chosen to combat this issue of interpretability and compare feature importance in MLP models. They help quantify how much each variable adds to the model’s outputs, ultimately aiding in deciding which features are, and are not, necessary. It is a common approach used when attempting to draw out explainability from complex machine learning techniques, such as neural networks. Much like odds ratios, how much more likely a positive outcome is to occur than a negative outcome when the variable in question increases in value, for regression models, plots using these values can help to visualise how different values within variables influence predictions (as shown by the key in Fig 8), as well as ordering the impact features have on the model’s outputs. Feature importance plots from the optimal *Premier League* and *Bundesliga* models (MLP and XGBoost, respectively) are displayed below.

For *Premier League* features (Fig 8), distance naturally dominates the graph, with low values significantly increasing probability predictions and high values decreasing them. Player value is deemed to be the 3rd most important feature, more influential than common xG model additions such as most shot types (head/body and weak foot) and all shot situation variables. In addition, many proxies for team quality are included amongst the most important features. Team’s average spend, team’s Elo score and previous season’s ranking placed 4th, 5th and 8th, respectively. This is most likely because, whilst it is widely considered to be an incredibly competitive competition, the majority of its titles have been won by teams from the so-called ‘Big Six’ (Arsenal, Chelsea, Liverpool, Manchester City, Manchester United and Tottenham Hotspur). Despite other team quality proxies showing expected trends in the impact its values have on the model’s outputs, the previous season’s ranking variable followed the opposite trend to what was predicted, with the implication that goal probability is increased the higher the ranking number (i.e., the lower they finished in the previous season’s table). This could be due to the fact that the order the ‘Big Six’ finish in can change significantly from season to season. Outside of this group, the positions of each team changed considerably within the same period. Also notable in Fig 8 is the disparity in effect on xG predictions between penalties and non-penalties. Whilst shots which do not occur from these situations naturally not altering model outputs, those taken from the penalty spot tend to add around 0.4 to estimates. However, its position can be explained by the fact that, in comparison to other match situations, shots originating from penalties are very rare.

**Fig 9:**
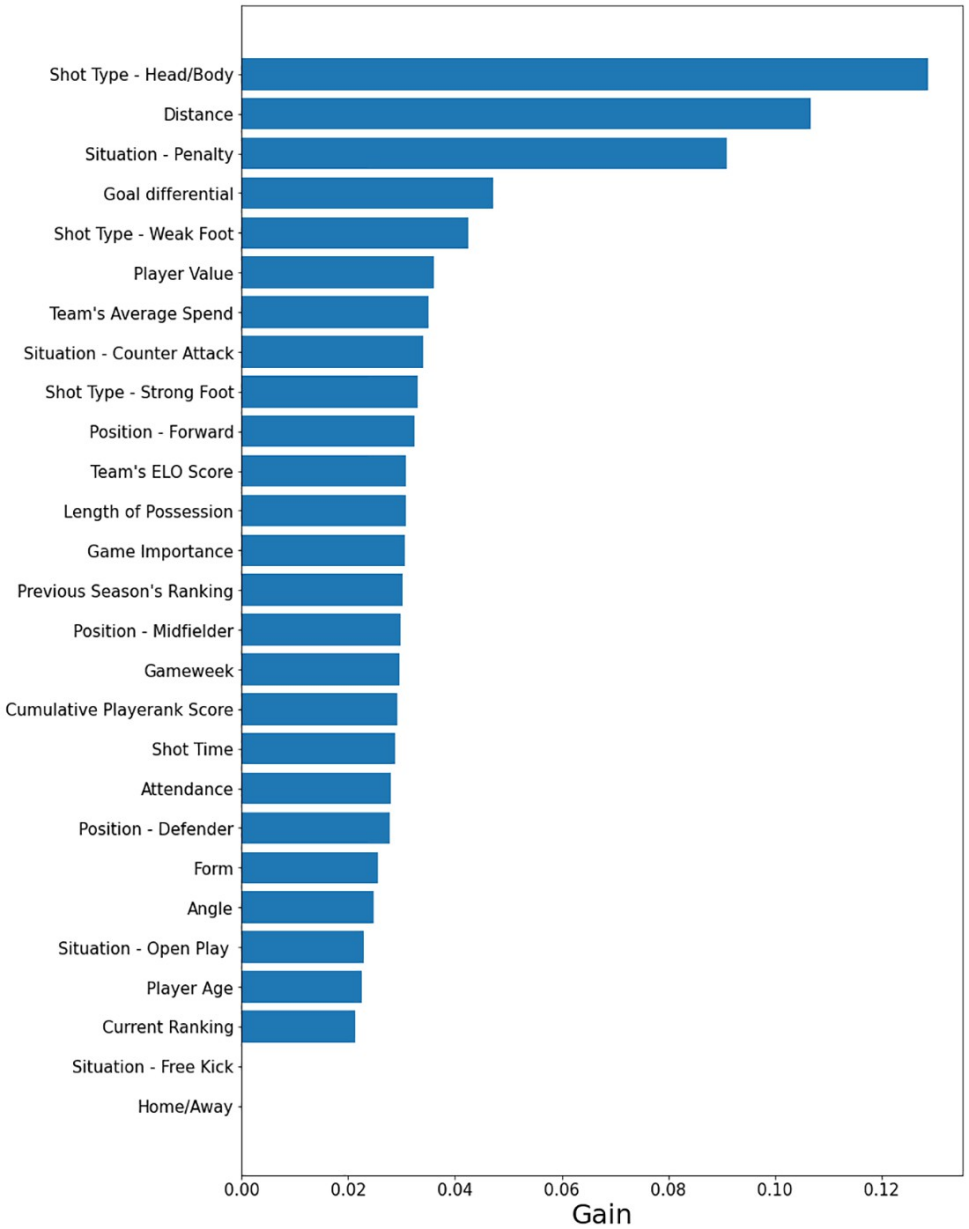
Important features for the German *Bundesliga* using the optimal model (in this case a tuned XGBoost model). We order the features in terms of Gain, the improvement in predictability attained by the variable from splitting the dataset.

Feature importance for the German *Bundesliga* is displayed in Fig 9. The usual inclusions in expected goals models populate most of the places within the top ten, with the binary variable indicating whether the shot was taken with the body/head or not surprisingly deemed more influential than distance. In fact, all shot types were shown to be impactful in determining xG values, more so than in other leagues. In addition to this, when analysing the odds ratios across all leagues, it was revealed that the value associated with forwards in the *Bundesliga* was by far the highest in any competition (1.2935). This could imply there are subtle tactical phenomena, such as an increased reliance on heading and/or greater onus on strikers to score goals, within this competition that either do not exist or are at least not as prevalent in others. Delving deeper into these odds ratio findings shows that the *Bundesliga* contains the two closest values for shots from a player’s strong (1.1797) and weak (1.1214) foot (a difference of just 0.0583), possibly meaning that two-footedness is more of a requirement, or at least more sought after, in this league compared to others. This plot also shows that some features not previously incorporated into expected goals models examined within the literature do rank relatively highly in importance. These include goal differential (4th), player value (6th) and team’s average spend (7th), further demonstrating the value of their inclusion.

Following results from other leagues, inferences can be made about their differing characteristics. Firstly, what is clear within the results from *Ligue 1* is that team quality in general has a strong impact on goal probability predictions (Elo Score 3rd, Current Rank 7th and Previous Season’s Ranking 8th). This is most likely the case due to the predictable nature of the competition in recent years. In four of the five seasons previous to the 2017–18 campaign, Paris Saint-Germain won the title and, within the same period, twelve out of the fifteen top three finishing positions have been occupied by either PSG, Monaco or Lyon. In Spain’s *La Liga*, PlayeRank scores placed surprisingly high (when compared to other competitions) due to the high variability in its entries resulting from the performances of Cristiano Ronaldo and Lionel Messi (widely considered the two best players in the world), alongside other players (e.g., Suarez, Griezmann and Benzema) who also had stellar seasons. This was why the La Liga’s odds ratio value for PlayeRank was the only one within the top five leagues to indicate that the higher the score, the more likely a shot is to be successful. Finally, in *Serie A*, the free kick situation and defender position variables place the highest out of all leagues. Furthermore, their odds ratio values were not surpassed by any of the other competitions’ counterparts (1.4062 and 0.92475, respectively), implying that free kicks and shots by defenders are most likely to result in a goal in the Italian league. This could be explained by a possible reliance on set pieces (direct/indirect free kicks and corners) in order to score goals within *Serie A*, in part, because it is usually in these situations that defenders are positioned close to the opposition’s goal and are therefore given the chance to shoot. The lowest odds ratio values for open play (0.41467) and counter-attack situations (0.58595) were found in this league, and that the latter variable was shown to add no benefit to the model’s xG predictions (with 0 gain).

One of the most intuitive factors which influences shot outcome but has not previously extensively been researched within the scope of expected goals is player ability. Two proxies were incorporated into the models built in order to test whether the factor had a significant impact on probability predictions: player value and cumulative PlayeRank score. Whilst cumulative PlayeRank score can be largely considered an unsuccessful addition to xG models, player value can certainly be deemed a successful one. Even though cumulative PlayeRank score ranked 4th for La Liga’s optimal model, it only ranked as high as 13th in all other leagues. Furthermore, inspection of the SHAP plots and odds ratios revealed that, for the most part, lower values of the feature resulted in an increase in goal probability estimation (the only exception is the odds ratio for La Liga, explaining its high ranking for the league’s optimal model). This is probably due to the fact that, since it accumulates values over a season, a large proportion of its entries will be close to zero (for matches earlier on in the season), regardless of the player’s quality. On the other hand, player value was shown to have a big impact on model outputs. It placed within the top ten feature importance plots for five out of the six leagues (and combination of leagues), placing 11th in the only other competition. Additionally, all SHAP plot and odds ratio results indicate that likelihood of scoring from a shot increases the more valuable the player is. This is in line with both what was expected and what was discussed in the Materials and methods section..

Another factor examined, similar to player ability but at a more macro-level, was team quality. Many proxies for differences in the success of football clubs were included at the modelling stage. These were form, team’s average transfer spend, team’s ELO rating, previous season’s ranking and current rank (ranking at time of match). These features had varying impact from league to league, making it difficult to judge whether they were valuable additions. This being said, form and current rank can be considered poor inclusions, with both placing below 20th (out of 27) in four out of the six model types. For current rank, this was also reflected in the SHAP and odds ratio values. For some leagues, they indicated the expected trend: an increase in goal probability if the entry is lower, for others, the opposite trend was observed. Despite the fact that previous season’s ranking performed better in terms of feature importance, it too suffered from the same problem (i.e., unclear trend) as current rank. Both team’s average transfer spend and team’s ELO rating generally ranked relatively high in feature importance. Form and current rank both indicate how a team is performing in recent matches, something which previous season’s ranking extends to the start of the given season. However, team’s average spend and team’s ELO rating are more so determinants of a club’s success over a long period of time. The differences in how much these variables help to predict goal probability suggests that the long term state of a given team matters more than the short term when it comes to quantifying team quality.

The proxies used to quantify psychological effect were match attendance, match importance and goal differential. Attendance can be considered unimportant (or at least less important than most other features), match importance can be considered medium-to-low in terms of impact on the models and goal differential can be considered an influential variable. Attendance places relatively low for the majority of leagues, whilst match importance ranks somewhere in the mid-range. SHAP plots and odds ratio results showed that, in general, goal probability increased both when there were fewer fans present at matches and when the consequences of match outcome were less crucial to the fortunes of a team. These findings possibly imply that psychological pressure can affect goal likelihood. However, this would require further research to determine whether the features were either of comparatively low importance or of low importance in general. Finally, goal differential was one of the most influential variables included in the expected goals models. Whilst it did place 11th and 13th for the Premier League and Ligue 1 respectively, which implies it is somewhat effective, it placed within the top ten for all other leagues (as high as 3rd). Furthermore, the SHAP plots and odds ratio results indicated that the less a team is losing by and the more a team is winning by, the more likely they are to score from a given shot.

### Predictive comparison results

To assess the predictive ability of expected goals statistics and traditional metrics, information on teams’ performances from the previous *x* matches (with *x* > 1). The reason for this is because, in order to attempt to predict future success or failure of teams with confidence, measure of how well the team has been doing in recent matches needs to be used as an input. For each of the top five leagues (and all leagues combined), outputs for goal probability of each shot from the optimal model were attributed to the matches and teams that they corresponded to. Additionally, xG values calculated by StatsBomb, an industry leader within football analytics, were assigned to their respective matches and teams. For each team within each league, data on their shots, goals, xG values computed in this study and xG values computed by StatsBomb from the previous six matches were summed and used as inputs (with some manipulation) into two separate models. A value of *x* = 6 was chosen following research from Baboota and Kaur [25] into the optimal value for their form variable.

The two models this information was used for involved predicting the team’s next match result (i.e., loss, draw or win) and estimating the team’s future goal ratio (goals per match) averaged over their subsequent six matches. For the latter, all inputs were altered to their mean over the previous six matches, rather than their sum. Since this was not a classification task, but instead concerned the prediction of a statistic’s value, linear regression was used to model outputs. A neural network (MLP) approach was chosen for loss/draw/win prediction. Evaluation metrics for classification tasks (accuracy, precision, recall and F1 score) and mean squared error (for regression) were employed to determine how well each statistic performed. Findings from these analyses are shown in Table 4.

**Table 4:** Test data results for comparison between expected goals statistic and traditional metrics.

League	Statistic	Next Match Result				Future Goal Ratio
		Accuracy	Precision	Recall	F1 Score	MSE
Premier League	Shots	0.4635	0.3809	0.4635	0.4035	0.2669
	Goals	0.4948	0.3916	0.4948	0.4296	0.2925
	Our xG model	0.5208	**0.4404**	0.5208	0.4538	**0.2013**
	StatsBomb xG	**0.53645**	0.4173	**0.5365**	**0.4689**	0.2207
La Liga	Shots	0.4635	0.3809	0.4635	0.4035	0.2669
	Goals	0.4948	0.3916	0.4948	0.4296	0.2925
	Our xG model	0.5208	**0.4404**	0.5208	0.4538	**0.2013**
	StatsBomb xG	**0.53645**	0.4173	**0.5365**	**0.4689**	0.2207
Bundesliga	Shots	0.4079	0.2978	0.4079	0.3365	0.2823
	Goals	0.375	0.2737	0.375	0.3164	0.2943
	Our xG model	**0.4737**	**0.3462**	**0.4737**	**0.3989**	**0.2310**
	StatsBomb xG	0.4079	0.2997	0.4079	0.3445	0.2843
Serie A	Shots	0.4079	0.2978	0.4079	0.3365	0.2823
	Goals	0.375	0.2737	0.375	0.3164	0.2943
	Our xG model	**0.4737**	**0.3462**	**0.4737**	**0.3989**	**0.2310**
	StatsBomb xG	0.4079	0.2997	0.4079	0.3445	0.2843
Ligue 1	Shots	0.474	0.3738	0.474	0.4172	0.3462
	Goals	0.4375	0.3518	0.4375	0.3826	0.3311
	Our xG model	**0.5104**	**0.4032**	**0.5104**	**0.4486**	**0.2757**
	StatsBomb xG	0.474	0.3737	0.474	0.4125	0.3605
All Leagues	Shots	0.474	0.3738	0.474	0.4172	0.3462
	Goals	0.4375	0.3518	0.4375	0.3826	0.3311
	Our xG model	**0.5104**	**0.4032**	**0.5104**	**0.4486**	**0.2757**
	StatsBomb xG	0.474	0.3737	0.474	0.4125	0.3605

**Fig 10:**
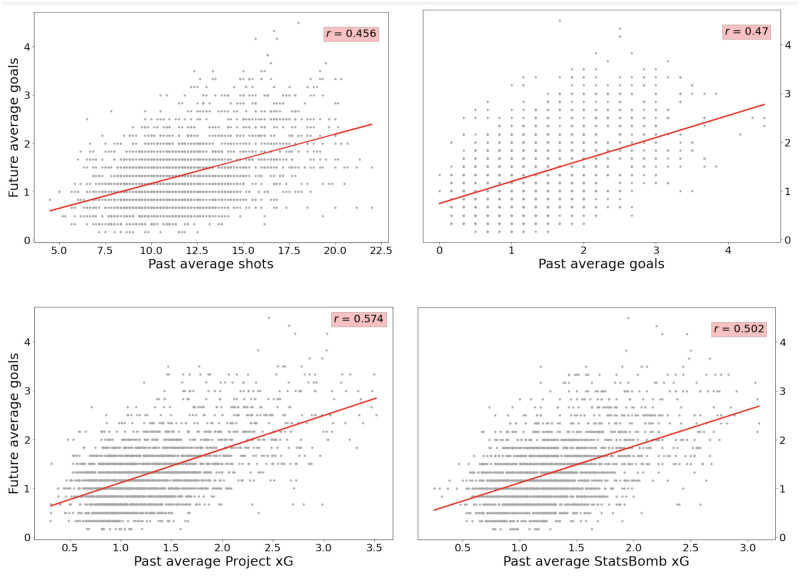
Statistics from all leagues data plotted against future average goals. The differences between each metric’s ability to predict future goal ratio are examined by plotting the best fit line through each statistic’s values (from all leagues combined) against average goals over the subsequent six matches and calculating Pearson’s *r* to deteermine their level of correlation.

In addition to mean squared error, differences between each metric’s ability to predict future goal ratio were examined both visually and numerically. This was achieved by plotting the best line of fit through each statistic’s values (from all leagues combined) against average goals over the subsequent six matches and calculating Pearson’s *r*, in order to quantify how strongly correlated two variables are. These results are displayed in Fig 10.

Results from comparing the xG values estimated in this study and those supplied by StatsBomb against traditional metrics (shots and goals) in terms of predictive ability show that the expected goals statistic is the superior indicator of a team performance. For all leagues analysed, expected goals is bettered in only one evaluation metric for one competition (precision in Serie A), with xG outperforming the other statistics in every other league and for every measure considered. Examination into differences between both expected goals statistics reveal that the xG values calculated as part of this study were generally better at predicting next match results, with those supplied by StatsBomb having higher accuracy scores for just two out of the six leagues. Additionally, at the more granular level, when analysing future goal ratio estimates, it is clear that the all leagues xG model in this study far outperforms any of the other metrics included, with it an average of 0.0235 away from the next best performing statistic in terms of mean squared error. This is also reflected in the plots in Fig 10. Whilst values for both shots and goals vary considerably from the best line of fit and therefore produce lower *r* coefficients (0.456 and 0.47), the all leagues xG model and StatsBomb model tend to cluster much closer to the curve, resulting in higher *r* coefficients (0.574 and 0.502). When comparing the plots for the latter two, it is evident that the StatsBomb model over-predicts medium-to-high past average values to a greater extent than the all leagues xG model in this study, perhaps leading to a much higher *r* coefficient for the latter.

One other notable finding from the next match result predictions was the inability for any of the leagues’ models to predict draws. This is a common problem in match outcome prediction [25–28], and is most likely being due to the fact that, whilst they are the least prevalent result, draws occur at a relatively high incidence rate (around 25%). Additionally, this result is interdependent on the performance of both teams playing, whereas wins and losses can frequently solely depend on the performance of one team (either playing well or poorly). No draws were predicted for the each and all leagues analysed. This then meant the values for recall and accuracy were the same within each model.

## Conclusion

The main motivations behind this project were to add to the limited pool of research into expected goals, to further improve the performance of xG through the addition of influential features to model goal probability and to consolidate the prevailing yet not unanimous view that the metric is of significant value to everyone within the football community.

The optimal model built in this project was shown to be competitive when compared to results from other studies within the existing literature. In addition to this, variables previously rarely considered or untested were examined at the modelling stage, providing new insights into factors which can influence goal probability. The application of these features onto separate football leagues has allowed for examination of the varying levels of impact they have on different competitions for the first time.

Results from building xG models incorporating these previously untested features showed that some proxies were deemed to be impactful within each league, some others had varying effects on probability predictions and some were found to be of little use in explaining the randomness in goals. The most important variables were player value (as calculated by the website Transfermarkt), representing differences in player ability, and goal differential, representing psychological effects during matches. Both of these indicated that the higher the value of the given feature, the more likely a shot is to result in a goal. Proxies for team quality (ELO ratings, average summer transfer spend, form, previous season’s final ranking and position in table at time of match) had differing influence on goal probability across leagues. For example, many of the above features placed highly in France’s *Ligue 1*, this most likely due to predictable nature of the competition, with Paris Saint-Germain winning the title in four of the five seasons previous to the 2017–18 campaign. Rankings of features in other leagues allowed for inferences to be made about their structure and these are detailed in the expected goals modelling results section. Despite the fact that some variables (length of possession, shot time, player age and gameweek) added to the models did not appear to significantly influence predictions, the multitude of findings described above demonstrate that this objective has been successfully met.

Finally, analysis into the predictive ability of traditional metrics against expected goals concluded with the latter outperforming the former in all areas of next match prediction (except for Serie A’s precision score on test data) and future goal ratio forecasting. This puts into numbers the true power within xG and demonstrates why it is ubiquitously referenced by analysts in football clubs and betting companies. In addition to this result, when looking into discrepancies between both xG sources, it was found that, in the majority of cases, the values generated as part of research into expected goals in this study were superior predictors to those collected from StatsBomb. This is visualised (and indeed further quantified) by both their correlation plots (and their *r* coefficients), which implies that future goal ratio best fits predictions made by the former xG model. These findings serve to explain why it is no wonder experts consider xG to be of such use in a variety of situations at football clubs, encompassing player development, team performance evaluation and player acquisition, amongst other key areas.

Whilst this study has produced impressive results and been rigorous throughout, it does have some limitations that could be tackled in further work.

The structure of some features included in expected goals models could have been improved. Firstly, a frequent drawback to calculating PlayeRank scores cumulatively was the large proportion of lower values within the variable. This was due to the simple fact that a footballer’s playing time was not taken into consideration. For example, a shot taken by a footballer playing incredibly well at the midpoint of the season would have a similar cumulative PlayeRank score to a shot taken by a footballer playing at an average level at the end of the season. This then led to most models predicting higher goal probabilities the lower this feature’s value. This variable was included as such to produce a feature whose values change throughout the season, according to how well the player it is attributed to is performing. This issue could be fixed by contextualising the PlayeRank score for a match within the gameweek is occurred in, possibly by dividing the value by its associated gameweek. Similarly, the approach to engineering the angle variable was not optimal. As the kernel density plot in Fig 2 shows, the distribution of angle values is bell-shaped. This is not a problem for neural networks and decision tree-based algorithms, both of which can capture non-linear trends within its features. However, one assumption of logistic regression is that the relationship between an independent variable’s log odds and the dependent variable must be linear. The structure of the angle feature within this study could violate this assumption, perhaps explaining why it surprisingly places relatively low in terms of feature importance. In order to change this, its values could be taken to be the angle between its nearest side of the goal line and the position the shot was taken, instead of the left side of the goal (when facing the goal) and the shots, *x*, *y* coordinates.

Furthermore, since newly considered factors had to be incorporated into the models in the form of proxies, some of what these factors represent could have been lost when these proxies were determined. For example, one of the most influential features within all models was goal differential. Whilst this variable was included at the modelling stage as a proxy for psychological effects during a match, it could have aspects within it which more strongly point it out to be a proxy for team quality. This feature was deemed to increase goal probability estimates, the higher its values were. However, due to the intuitive fact that more successful teams tend to be in front in matches more often than less successful teams, the extent to which goal differential represents psychological effects can be put into question. To accommodate for this, other proxies for the same factor possibly affecting goal probability can instead be examined further or the goal differential values could be adjusted for changes in team quality.

We have included several overlooked variables in calculation of expected goals to produce a better prediction. It is however, not a complete measure of the predictability of a game’s outcome. We have not explicitly modelling expected shots on target. In spite of these limitations, the results produced in this study, alongside the statistic’s growing propagation within football, prove that expected goals can bring great value to analysts, pundits and fans alike. This goes to show why xG plays a key role in managing financial and tactical risk within a sport which is heavily influenced by randomness, allowing clubs to better forecast what is to come and safeguard their future.

## Supporting information

S1 Table Overview of features included in expected goals models. The first 4 features are categorical features taking the values listed, the rest are numerical values in the ranges shown.
(PDF)
