# Supplementary material for: Expected goals in football: Improving model performance and demonstrating value
Source: PLoS One. 2023 Apr 5;18(4):e0282295. doi: 10.1371/journal.pone.0282295 (PMC10075453; doi:10.1371/journal.pone.0282295)
Supplement: S1 Table — The first 4 features are categorical features taking the values listed, the rest are numerical values in the ranges shown. (PDF) [file pone.0282295.s001.pdf]

S1 Table. Overview of features included in expected goals models The first 4 features are categorical features taking the values listed, the rest are numerical values in the ranges shown.

Feature	Values	Description
Side	Home, Away	Whether the player taking the shot is playing at home or away
Body Part	Head/Body, Strong/Weak Foot	What part of the body is used to take the shot
Match Situation	Open Play, Counter, Free Kick, Penalty	Which type of situation the shot occurs in
Position	Defender, Midfielder, Forward	What position the shot-taker plays in
Gameweek	$[1, 38]$	How far through the season the match in which the shot occurs is played
Time of shot	$[1, 6190]$	The second of the match in which the shot occurs
Goal Difference	$[-8, 8]$	How many goals the shot-taker's team is leading or losing by
Length of Possession	$[0, 398]$	The length of time (in seconds) the shot-taker's team has sole possession of the ball before the shot occurs
Age	$[15, 41]$	The age of the shot-taker on matchday
Distance	$[1, 101]$	The Euclidean distance from the centre of the goal line at which the shot is taken
Angle	$[0, 180]$	The angle (in degrees) between the left side of the goal line and the location at which the shot is taken
Current Rank	$[1, 20]$	The league position the shot-taker's team occupies before the match is played
Previous Season Ranking	$[0, 20]$	The league position the shot-taker's team finished at the end of the previous season
Player Value	$[0, 180000000]$	The shot-taker's Transfermarkt value
Average Transfer Spend	$[0, 107100000]$	The average summer transfer fee per player (including loan fees) of the shot-taker's team
Cumulative PlayeRank Score	$[-0.6, 1.7]$	The shot-taker's cumulative PlayeRank value at time of match
Match Attendance	$[0, 97939]$	The attendance figure at the match in which the shot is taken
Match Importance	$[0, 1]$	How important the result of the match is to the shot-taker's team
Team Form	$[0, 1]$	How the shot-taker's team has been performing in recent matches
Elo Rating	[§114, 2088]	The Elo rating of the shot-taker's team on the day of the match

Table 1. Overview of features included in expected goals models. The first 4 features are categorical features taking the values listed, the rest are numerical values in the ranges shown.
